# “It's Not a One Size Fits All”: Service Users’ Views on How Service Structure Shapes Quality of Abortion Care in Britain. Findings from the SACHA Study.

**DOI:** 10.1111/sifp.70058

**Published:** 2026-07-13

**Authors:** Rachel Scott, Maria Lewandowska, Rebecca Meiksin, Natasha Salaria, Patricia A. Lohr, Sharon Cameron, Jennifer Reiter, Melissa Palmer, Rebecca S. French, Kaye Wellings, the SACHA Study Team*

## Abstract

Abortion care in Britain has undergone significant transformation in recent years, with increasing use of medication, home management, and telemedicine. However, provision remains predominantly through specialist standalone clinics, particularly in England. Drawing on the interlinking frameworks of abortion exceptionalism, stigma, and quality of care, we analyzed 48 qualitative interviews with people who recently had abortions to explore how service‐users perceive different models of care in terms of access, continuity, holistic care, and the normalization of abortion. Participants highlighted the importance of both *place* (standalone vs. integrated) and *provider* (specialist vs. generalist). Primary care was seen as convenient and supportive of continuity (e.g., for post‐abortion contraception) and normalization of abortion as routine healthcare. Specialist clinics, however, were valued for expertise and nonjudgmental care. Community sexual and reproductive health services offered a blend of these benefits. Preferences varied depending on individual circumstances, pointing to the need for flexible, person‐centered care. This study contributes to ongoing discussions about how best to organize abortion services to support reproductive autonomy and equity. Importantly, structural change alone is insufficient; attention to the ongoing experience of stigma remains essential to delivering high‐quality, person‐centered abortion care.

## BACKGROUND

Under the terms of the 1967 Abortion Act, abortion in Britain must be authorized by two medical practitioners, provided by a registered medical practitioner, and carried out in a place approved by the Secretary of State (Department of Health and Social Care 2025; Public Health Scotland 2024). While this legal framework established abortion as a lawful medical intervention, its specific requirements have contributed to a long‐standing separation of abortion services from mainstream healthcare. In England and Wales, this separation manifested in the emergence of independent and charitable clinics that filled early gaps in NHS provision (Davis et al. 2019; Sheldon et al. 2019), a pattern which persists today. In 2022, 80 percent of abortions in England and Wales were delivered by the independent sector under the NHS contract, although the vast majority (98 percent) are commissioned and funded by the NHS and so free at the point of care (Department of Health and Social Care 2025). Scotland, in contrast, retained and expanded services in the NHS, including through community sexual and reproductive health (SRH) clinics (Cameron et al. 2016), with 99 percent of abortions provided by the NHS in community SRH services or hospital gynecology departments (Public Health Scotland 2024).

This structural division, whereby abortion has primarily been provided by specialist healthcare practitioners in specialist clinical services, rather than by generalists within primary care or integrated as part of mainstream healthcare services, has been conceptualized within the framework of abortion exceptionalism—the idea that abortion is regulated differently from, and more stringently than other procedures of similar frequency and safety when there is no clinical basis for doing so (Serpico 2021). Abortion exceptionalism extends beyond legal regulation to encompass how care is provided (Joffe and Schroeder 2021). It is argued that the independent‐sector model of care by separate independent providers and often on separate premises, perpetuates this idea that such services are “unusual, different or in some way out of the norm” (Emmerich 2023, 2) and contributes to the stigmatization of abortion by reinforcing the idea that it is not an ordinary part of reproductive healthcare and producing the clinic setting as a site of abortion stigma (Footman 2025; Sorhaindo and Lavelanet 2022).

This research also draws on sociological theories of stigma, particularly Goffman's foundational work (1963) and subsequent critiques and elaborations by Link and Phelan (2001, 2014) and Tyler and Slater (2018) that conceptualize stigma as a process embedded in power relations, enacted through labelling, stereotyping, and institutionalized discrimination. Tyler and Slater remind us that stigma can be used as a technique of governance, that is, a way that institutions and systems of power produce and reproduce inequality. In the context of abortion, stigma may be produced not only through individual attitudes but also through systemic factors like legal regulation, funding arrangements, and the design and delivery of services. The separation of abortion from mainstream healthcare can signal marginality, reinforcing stigmatizing discourses (Footman 2025).

The spatial and institutional organization of abortion care, which both shape and are shaped by discourses of abortion exceptionalism and stigma, have implications for the quality of care experienced by those accessing abortion services. Quality of care is a concept which has long been recognized as multidimensional, encompassing not just clinical safety but also interpersonal and structural components (Darney et al. 2018). Judith Bruce's (1990) framework on the fundamental elements of quality of care in reproductive health sets out six interrelated domains: choice of method, information provided, technical competence, interpersonal relations, follow‐up and continuity mechanisms, and the appropriate constellation of services. It has since been built on by others (e.g., to explicitly encompass access), and its key principles remain influential in informing reproductive health guidelines and evaluations (e.g., World Health Organization Abortion Care Guidelines 2022). The structural organization of abortion services, including whether services are integrated into or provided separately from mainstream healthcare, might impact multiple domains of quality of abortion care including access, continuity and follow‐up, technical and interpersonal skills of the provider, and whether services are organized in a way that is convenient and acceptable to those seeking care (Maxwell et al. 2021; Mazza et al. 2022; Wulf et al. 2023). Abortion exceptionalism and stigma can therefore be considered as intersecting with both how services are organized (configuration of care) and how they are accessed and experienced by users (quality of care).

Significant therapeutic, technological and regulatory changes have occurred since the law on abortion was introduced in Britain in 1967. Medical abortion, involving administration of two sets of pills, mifepristone and misoprostol, is now a mainstay of provision and has transformed abortion care (Berer and Hoggart 2018). The proportion of all abortions in Britain that were medically induced nearly doubled between 2010 and 2020, and in 2022 accounted for 86 percent of the total number in England and Wales (Department of Health and Social Care 2025), and 98 percent in Scotland (Public Health Scotland 2024). Advances in technology have facilitated remote support for abortion, obviating the need for attendance at clinical facilities for many. In 2022, temporary legislation to allow both mifepristone and misoprostol to be taken at home, occasioned by the COVID‐19 pandemic in 2020, was made permanent and that year more than 60 percent of all abortions were home‐managed (Department of Health and Social Care 2025; Public Health Scotland 2024).

The changes have implications for where abortion is provided and by whom, and, as the location and mode of abortion provision evolve, the structural organization of services, and how this shapes quality, merits closer scrutiny. Clinicians in both low‐ and high‐resource settings recommend integrating abortion into mainstream healthcare services, including primary care, as an essential part of reproductive healthcare (Dragoman et al. 2022). The World Health Organization suggests that all cadres of health workers may be trained to provide comprehensive abortion care, and this is held to improve quality of care, including access (World Health Organization 2022).

Evidence from other countries, including the United States, Canada, Australia, and Sweden, shows abortion provision in primary and other nonspecialist settings to be safe for patients, acceptable to healthcare practitioners and cost‐effective (Dutton‐Kenny et al. 2024; Kaller et al. 2022; Lai and Cohn 2024; Sjöström et al. 2016; Srinivasulu et al. 2024; Tressan et al. 2024; Zusman et al. 2023). Provision in primary care is seen as offering benefits in terms of convenience and improved access—particularly in rural areas (LaRoche et al. 2022; Lerma, Coplon, and Goyal 2023; Mazza et al. 2022; Yanow 2013; Zusman et al. 2023), and as allowing continuity of care through engagement with a wider range of health services (Dragoman et al. 2022; Wulf et al. 2023). Incorporation into primary care is held to facilitate provision of more personalized and holistic care (Mazza et al. 2022; Yanow 2013; Wulf et al. 2023). Within Britain, Scotland's model of NHS‐based abortion provision through community SRH services is reported to improve access to comprehensive contraceptive and sexually transmitted infection care (Cameron et al. 2016, 2017). Finally, embedding abortion care into the familiar spaces of primary care settings is hypothesized to reconceptualize abortion services as essential reproductive healthcare (Dragoman et al. 2022), thus contributing to normalization of abortion.

Integrating abortion provision into primary care settings has been proposed as potentially impacting multiple dimensions of quality in abortion care (Dragoman et al. 2022; Maxwell et al. 2021; Mazza et al. 2022; Wulf et al. 2023; Zusman et al. 2023). However, most empirical research on the merits of integrating abortion provision into wider healthcare services has examined the perspectives of healthcare professionals (Dawson et al. 2017; Haas et al. 2022; Lerma, Coplon, and Goyal 2023), with relatively little attention paid to service users’ perspectives (Footman 2025). In this study, we address this gap by exploring how people with recent experience of abortion perceive the relative merits of integrated compared to standalone provision. Drawing on the interlinking frameworks of abortion exceptionalism, stigma, and quality of care, we examine perceptions of how service structure shapes experiences of access, continuity of care, provision of personalized and holistic care, and normalization of abortion.

## METHODS

*Sampling*: A purposive sample of those with recent (past 2–8 weeks) experience of abortion was recruited between July 2021 and August 2022 from six sites in Britain: three independent‐sector services commissioned by the NHS (a telemedicine hub in the North of England, and in‐person clinics in London and Southwest England); and NHS sites in Scotland (an SRH service), Wales, and England. Eligibility criteria were: age 16+ years, ability to give informed consent, UK residence, and abortion for reasons other than fetal anomaly. To ensure diversity in the sample, the profile of the recruited sample was regularly reviewed, and underrepresented groups were specifically targeted. Clinic or telehub staff introduced the study to patients seeking an abortion who had consented to treatment. Options for initiating participation included speaking to a researcher on site, making direct contact themselves, or, with permission, having their details passed to the researchers to follow up.

*Data collection*: Semistructured 60–90 minute interviews were carried out by phone or videocall and, with permission, were audio‐recorded and transcribed. The Participant Information Sheet and Consent form were shared with participants prior to the interview, and participants had the opportunity to ask questions and seek clarification on any aspect of the research prior to taking part. Verbal consent to participate in the study was recorded in the interview. A £20 high‐street voucher was offered in appreciation for their time. Participants were invited to reflect on their actual experience of abortion provision and also to consider the respective merits of different models of care, including provision in standalone abortion services and in primary care settings like general practice, pharmacies, and community SRH services.

*Data analysis*: Data were analyzed using the framework method, a method that is suited to answering service‐delivery and policy‐related questions and allows both deductive data analysis based on predetermined themes and inductive analysis of additional themes identified in the data (Gale et al. 2013). An initial matrix was created into which summary data were entered, by case and by code. Textual data relevant to the research question were identified and selected for thematic analysis and independently coded by RS and KW. We drew on potential impacts of integrating abortion into wider healthcare on aspects of quality of care identified in the existing literature to deductively create a coding frame, that is, ease of access, continuity of care, provision of holistic care, and normalization of abortion. Additional themes were drawn inductively from participants on their own experiences and their interpretations of what alternative models of abortion care might offer, drawing on their broader experiences of healthcare.

*Ethical statement*: This work was approved by LSHTM (Ref: 22761), NHS (IRAS Project ID: 291993), and BPAS (Ref: 2021/02/WEL). All participants gave informed consent prior to enrolment in the study.

## RESULTS

The 48 participants were aged 16–43. Thirty‐nine had had a medical abortion, eight a surgical abortion, and one both (unsuccessful medical abortion, followed by surgical abortion). Characteristics of the participants are presented in Table 1.

**TABLE 1:** Sample characteristics of participants included in analysis. Medication abortion is abbreviated to MA

ID	Abortion method	Age	Ethnicity	Children	Previous abortions	Abortion location
01	Home MA	31–35	White British	No	No	England
02	Home MA	26–30	White British	No	No	England
03	Home MA	21–25	British Bangladeshi	No	Yes	England
04	Home MA	26–30	White British	No	No	England
05	Home MA	36–40	White British	Yes	Yes	England
06	Home MA	36–40	White British	Yes	Yes	England
07	Home MA	41–45	White British	Yes	Yes	England
08	Home MA	31–35	White British	No	Yes	England
09	Home MA	21–25	White British	No	No	Wales
10	Home MA	31–35	White British	No	Yes	Scotland
11	Home MA	21–25	White British	No	No	Scotland
12	Home MA	26–30	White Irish	No	Yes	England
13	Home MA	21–25	White British	No	No	England
14	Home MA	26–30	White British	Yes	No	Scotland
15	Home MA	31–35	White British	Yes	No	Scotland
16	Home MA	31–35	White Polish	No	No	Scotland
17	Home MA	26–30	White British	No	No	Scotland
18	Home MA	36–40	White Canadian	No	No	Scotland
19	Home MA	21–25	White British	No	No	Scotland
20	Surgical	36–40	Not specified, Hungarian	Yes	Yes	England
21	Home MA	36–40	Not specified	Yes	No	Scotland
22	Home MA	26–30	White British	No	Yes	Scotland
23	Surgical	26–30	White British	No	Yes	Scotland
24	Home MA	26–30	White British	No	No	Scotland
25	Home MA	26–30	White British	Yes	Yes	England
26	Surgical	21–25	White British	No	No	England
27	Surgical	31–35	White British	Yes	No	Scotland
28	Home MA	26–30	White Hungarian	No	Yes	Scotland
29	Home MA	21–25	Pacific Islander	No	No	Scotland
30	Home MA	31–35	White British	No	No	England
31	Home MA	36–40	White British	No	Yes	Scotland
32	Home MA	16–20	White British	No	No	Scotland
33	Home MA	31–35	White British	Yes	Yes	England
34	Home MA	36–40	White British	Yes	Yes	England
35	Home MA	31–35	White British	Yes	No	England
36	Surgical	36–40	Black African	Yes	No	England
37	Home MA	21–25	White British	No	No	England
38	Home MA	16–20	White Asian	No	No	England
39	Home MA	21–25	White British	No	No	Wales
40	Surgical	31–35	Asian Nepali	Yes	Yes	England
41	Surgical	21–25	White German	No	Yes	England
42	Surgical	31–35	Black British	No	No	England
43	Home MA	16–20	Asian Afghani	No	No	England
44	Home MA	16–20	White British	No	No	Wales
45	MA at hospital	21–25	White British	No	No	Scotland
46	MA at hospital	16–20	White British	No	No	Scotland
47	Home MA	16–20	White British	No	No	Scotland
48	Home MA (failed) + surgical	21–25	Middle Eastern	No	No	England

### Access to Care

Most participants had obtained abortion medication from a standalone abortion clinic or community SRH service. When asked about the possibility of doing so from a wider range of health settings, like a GP or pharmacy, these were perceived as offering greater convenience in terms of the ease with which they were able to reach the service:

… your GP will be something near your house, it would be good to just like go to them [43]

… if it was available at the pharmacy then yeah, 100 percent.…if I had to do it again, and I could just grab it at the pharmacy… Definitely. [11]

Views on how speed of access would be affected if supplies of abortion medications could be dispensed in these services were more mixed. Some hypothesized potential advantages of care from their general practice or pharmacy, suggesting that *“You would probably get seen a lot quicker”* [41]; *“In terms of quick and easy and available then I would say probably it is going to a pharmacy”* [31]; “*I think it'd be a lot faster with the GP*” [45]. Others had concerns that the time factor might be an issue: *“Trying to get hold of your doctor can be a bit of a problem”* [11]. Several participants shared the view that time available for consultation might be a problem in primary care or that speed might be achieved at the expense of other aspects of quality of care, especially in general practice, for example,

I sometimes feel like GPs just want to see you as quick as possible. I feel they would just pop [the medication] in a bag, here are your instructions, kind of on you go [11]

These divergent views reflect the complex interplay between perceived ease of access and concerns about other aspects of quality care—tensions that underscore the challenges of provision within stretched primary care systems.

### Continuity of Care

Reports from participants’ experiences suggested that the potential of providers in standalone abortion services to provide engagement with the full range of health services had been limited. Points at which continuity of care was disrupted had tended to occur at the intersection of different healthcare services and sometimes resulted in services not being taken up. Frustrations and sometimes delays in obtaining contraception appeared to have been common. Describing efforts to have an IUD fitted following the abortion, one participant described a circuitous journey through different agencies.

Interviewer: And then when you actually did, get a copper coil fitted, was that at [independent abortion] clinic?

Participant: No. It was a bit of a nightmare actually, to get done. So the way the NHS works at the moment is, there is no sexual health clinic in my area, so I had to travel to [X Hospital] in [Y city]. [35]

Problems of continuity of care were most evident in the accounts of participants with complex health conditions. Among patients with conditions that necessitated care in a hospital, delays were caused by having to negotiate the interface between different services. A participant whose heart condition prevented her from having a medical abortion at an independent sector service described the consequent delays:

…at the appointment, [independent provider] said, “because you have a [heart condition] we can't do your abortion”. And I was like, that's really stressful. Can you refer me to the hospital? …“Yeah, yeah, we can refer you”. And I was like, Cool. How long does that take? And they were like, An average of three weeks…. But we have to scan you first and our next scan appointment isn't for two and a half weeks, and they might not accept your referral without a scan. [45]

When asked whether there were aspects of her experience that she saw as needing improvement, this participant replied, *“just better linking between these private charities and the NHS because they are definitely not in tandem at all … What I really don't understand is why the GP can't prescribe the medication.”*

Although streamlining of services was seen by many as needing improvement, it was notable that few were confident that provision integrated into mainstream healthcare would have guaranteed a smoother journey.

### Provision of More Personal and Holistic Care

We were interested in hearing whether the aim of providing more holistic care, combining physical care with personal and emotional support, was seen as better served by generalist or specialist services. We considered a standalone abortion clinic to be, by definition, a specialist service, while primary care settings might be generalist or specialist. Community SRH services, for example, have specific specialist expertise, while general practice and pharmacy‐based care would be considered generalist, addressing a broad range of health needs. In terms of meeting the needs of the whole person, some speculated on the advantages of receiving abortion care and support in primary care because of familiarity with the patient and their health records: *“they know your medical history and everything about you…”* [43]; *“For me, personally, I would prefer to go to the GP because the GP already has all of your health records”* [45].

Others cautioned that this may not be considered a benefit by everyone:

P: If you're going to your GP … you might have a doctor, a nurse who knows you … so just give an extra bit of comfort about mental reassurance throughout the process.

I: So, for you, the familiarity would*'*ve been an advantage?

P: Yes. But I think it would be different for other people depending on the stigma. [12]

… that depends… GPs would know them and know their families and that kind of thing, then yeah, that would be uncomfortable for people. [24]

Several participants valued the specialization offered by standalone abortion services. They were deemed to have advantages in terms of offering both technical and emotional support since that was the main focus of their work.

… when you're in your GP you're ringing for any number of reasons, it's not every day they deal with somebody who's inquiring about an abortion. … whereas these people that is what they're doing day in day out. [30]

…people [at independent provider] are trained … They deal, on a daily basis with people having a termination and know how to speak to people and deal with people [25]

Having personal and emotional needs met was particularly important to those who had experienced their abortion as a significant event, described by some as “massive” or “overwhelming.” This aspect of care had reportedly been well‐met in standalone services, and some had misgivings about whether it would be matched outside of these settings. Views on the possible impact of abortion provision within primary healthcare were largely hypothetical, but some who had first approached their general practice provided insights from that experience, as did others from their general impressions of GP care.

I: If you were able to approach your GP for abortion care would you have done that?

P: I think I did try to but they weren't very helpful. So, yeah I would. That was my natural go to. Yeah, I would if they would actually help me [42]

In theory, yes, …but I think maybe there would need to be a bit of … training on like, being them being sensitive. Because in my experience, I think a lot of GPs are a bit dismissive in a way [32]

Opinions varied, however, with the primary care setting in question. Community SRH services were seen by many as meeting needs of both specialist clinical and emotional support, demonstrated in the following participant's experience obtaining an abortion in such a service.

P: I think it would be better going to the [SRH clinic]… because that's what they specialise in… I feel like that's there for a reason, and that's what they've all learned to do is deal with that. …I felt good when I was there, and I felt comfortable there.

I: So is your answer more related to their clinical expertise or their attitudes or both?

P: I'd have to say both, I think. Yeah. [46]

These accounts suggest that for many abortion care is experienced not only as a medical procedure but also as a highly relational interaction, in which provider attitudes are considered as important as clinical training.

### Normalizing Abortion Care

Participants reflected extensively on the potential for integrating abortion care into primary care settings to contribute to normalizing abortion care, both in relation to destigmatizing abortion and, relatedly, in reconceptualizing abortion as no different to other healthcare procedures.

Few reported having experienced stigmatizing treatment in standalone services, despite, in many cases, having expected to. *“I thought I would be judged and stuff, and I really wasn't”* [25]; *“… the staff were really kind, I didn't feel judged”* [41]. Nor did participants report feeling stigmatized in community SRH settings: *“…they were nice and caring. I didn't feel embarrassed at all or ashamed about it”* [46].

Views on how providing abortion in general healthcare services would affect experience of stigma varied. It was noteworthy that where participants had encountered negative attitudes, they had tended to stem not from the specialist health professionals directly involved in their abortion care, but from auxiliary or reception staff.

I walked into [Hospital X] and explained the situation to the receptionist… She was like, “Well, we don't do abortions in this hospital…”. She was so rude about it. [45]

Feeling stigmatized was seen by many as less likely in specialist abortion or SRH services. In explaining this, participants reflected on provider attitudes and the experience of being in a setting with others attending for a variety of healthcare needs.

….But with a GP, just because they have their own judgements. Like it's not like you decide [to work] there because you support abortion or support the idea of a woman's choice. …It's not what the main aim of their profession is. [28]

… this [community SRH clinic] is kind of like a judgment‐free zone. …whereas when you go to a GP clinic, everyone's there for different things. The old woman who's got a cough will be wondering what they're doing …a GP is anybody and everybody with different things. [24]

The physical features of the healthcare facility also influenced perceptions of stigma. From one perspective, providing abortion in a separate location marked it off as in some way different, and so was considered likely to contribute to stigmatization.

I don't get why abortion needs to be put in a whole separate sphere. Maybe due to kind of historical kind of stigma around it and why it needs to be treated, you know, a little bit like, at arm's length compared to everything else …[13]

Some noted that access to abortion should be no different to access to other healthcare procedures, implicitly rejecting the notion of abortion exceptionalism in terms of its provision:

I think it should be as easy as me being able to make an appointment at my doctors for a smear test … I think it should be as easy as getting antibiotics. [13]

I don't see any reason why [medical abortion shouldn't be available in a pharmacy], I mean you can go get Viagra at a pharmacy, without prescription, I'm sure so, yeah, why make it harder? [22]

Other responses suggested that expectations and connotations of the clinic setting itself shaped their experience of abortion. They noted that if abortion could be provided in general practice or pharmacy settings, which were more familiar and which were associated with routine care, these features had the potential to moderate the experience in a positive way.

…if it was integrated into GP clinics, it will be far more approachable I think. [13]

Maybe [at a GP I would have felt] a little bit less intimidated. I was going into this specific building, and it is quite a big building as well that I went into and I was like “oh god, what is going to kind of happen here” [11]

… I feel like going to the clinic, it's very, it's that like one step up from a pharmacy, it feels very, like serious and very medical… if you're like used to go to your …pharmacy, maybe you feel a bit more comfortable there [32]

On the other hand, advantages of specialist services were also recognized. One participant described the reassurance she gained from being in the waiting room for her abortion at a community SRH clinic, perceiving that others were likely attending for the same reason.

…it was quite relieving to see that there were other girls here probably for the exact same thing [11]

Others saw the nonjudgmental, supportive care they had received at a specialist service (a standalone abortion clinic or a community SRH service) as contributing to making their experience feel normal, relating this term to treatment for other healthcare, and reassurance: *“everybody was super respectful and …yes, normalized it, it was just like any other medical procedure”* [22]. *“Everyone I talked to …was just so supportive, … and made me feel like reassured”* [32]. Some expressed uncertainty as to whether they would receive the same in nonspecialist settings.

… I felt like everyone that I spoke with [at the abortion clinic] was very sensitive to the situation… I don't know. Maybe would a GP be so aware? I don't know, but I guess yes [12]

### The Need for Choice

Across themes, participants returned to the importance of offering a range of options rather than pursuing a singular model of care. Although some participants unequivocally stated a preference for a particular model of care, they more commonly saw advantages and disadvantages of each. Recognition that needs and preferences in terms of care varied with different people and at different stages in life prompted circumspect opinions.

… there may be some women who find the whole process too intimidating, or too kind of like, overwhelming and therefore they might need a separate area to go to. … I don't think that then means that … every single abortion service needs to have its own separate place. I think that can be mix and match [13]

Everyone is in different circumstances… I think, having the flexibility, because it's not a one size fits all approach… to have the option for the woman to choose the way they prefer might actually be more beneficial. You know, they have a bit more control of the experience [9]

The need to take account of the needs of different people in different circumstances dissuaded many from taking a hard and fast position. Instead, emphasis was placed on ensuring the availability of different options: most participants expressed a desire for flexibility, autonomy, and the ability to choose the care setting that best met their needs at a particular point in time.

## DISCUSSION

To our knowledge, this is the first study in Britain to explore in‐depth the perceptions of people with experience of abortion on the relative merits of abortion provision in standalone services as compared to integrated into primary care. We interpret the findings focusing on perceptions of quality of care and through the lenses of abortion stigma and abortion exceptionalism—two interlinked theoretical frameworks that help illustrate both the structural configuration of abortion services and the tensions within and between participants’ accounts of how service configuration shapes different aspects of quality of care.

Participants’ reflections exposed complex views on different models of care and revealed the importance of two intersecting aspects of provision in shaping preferences—*place* (standalone vs. integrated into primary care settings) and *provider* (specialist vs. generalist). Participants recognized potential benefits of integrated care in terms of convenience and holistic support. However, they simultaneously expressed concerns that integration might come at the cost of expertise and quality of interpersonal care—elements strongly associated with specialized services (including community SRH). Challenges were identified for continuity of care in standalone services, especially for contraceptive provision postabortion and among patients with coexisting health conditions requiring greater engagement with other healthcare services. Some participants were doubtful that integrating abortion into primary care would alleviate this.

Participants' accounts showed that models of care shaped quality of care not only in terms of access to but also the experience of abortion. Participants’ perceptions were often underpinned by experiences or, more often, expectations of stigma. Link and Phelan (2001, 2014) and Tyler and Slater (2018) conceptualize stigma as an active process that is embedded in power relations and used as a technique of governance, including by institutions and providers through the way that services are designed and delivered. Our findings highlight how, on the one hand, integration into primary care held promise for destigmatizing abortion by repositioning it as part of legitimate, essential reproductive healthcare, as opposed to standalone services that might exacerbate stigma by exceptionalizing abortion and operating as a site of stigma. On the other hand, standalone services were seen as actively countering stigma through specialist providers’ nonjudgmental, affirming and supportive attitudes. That participants held these two “truths” at the same time illustrates how what optimal abortion care looks like is shaped by its exceptionalized and stigmatized status within health systems and society more broadly.

No single model of care was considered universally ideal; preferences depended on individuals’ unique needs, circumstances, and values, highlighting the central importance of patient choice in abortion care provision.

Participants’ reflections on models of abortion care and their potential impacts on quality of care complicate a dominant narrative found in professional discourse, which largely frames integration into primary care as favorable (Dawson et al. 2017; Haas et al. 2022; Lerma, Coplon, and Goyal 2023). Our findings suggest a more nuanced reality: perceptions of advantages and disadvantages of integrating abortion into primary care settings were more equivocal, and the consensus as to how service configuration shapes quality of care was less clear. Participants distinguished less between integrated and standalone provision, with responses instead focusing on the intersection of provider and place. Differences of opinion were expressed not only between participants’ accounts but also within them.

Comparison of our findings with those of other researchers is limited by the scarcity of similar studies and by differences in methodology and health system setting. Patient surveys in the United States comparing provision of abortion in primary care and in abortion clinics broadly indicate support for integration (Godfrey et al. 2010; Logsdon, Handler, and Godfrey 2012; Rubin et al. 2009; Srinivasulu et al. 2024; Tressan et al. 2024). Some of the reasons given for endorsement of different models in these studies—better continuity of care in integrated services, higher levels of skill and expertise in specialist clinics—are broadly consistent with our findings. Others—for example, more timely access—are more discrepant with our findings and highlight the importance of considering the social, economic, and health system context in determining optimal models of care. The constraints under which primary care practitioners in the NHS operate may help to explain the disparity between our findings and those of others (Pereira 2021).

Although limited research in Britain has directly compared views on different models of abortion care, findings from studies investigating access to and experience of abortion in this setting have found limitations in continuity of care among people with experience of abortion from independent providers (Footman 2024). Our findings align too with research that highlights interpersonal aspects of quality of care—including respectful treatment and emotional support—as central to the abortion experience for some (Altshuler et al. 2017; Hoggart et al. 2024; Lewandowska et al. 2024). In our study, participants valued the nonjudgmental approach of specialist abortion providers, and some doubted whether generalists could replicate this. Similar concerns have been raised by US providers (Karlin and Joffe 2023), although others in that study questioned the assumption that only specialists could provide person‐centered abortion care. Research in the United Kingdom adds further nuance to this picture, highlighting that the kinds of support needed are not necessarily best provided by health professionals, and the role of support from social and familial networks, talk lines, and online forums (Hoggart et al. 2024; Lewandowska et al. 2024).

While integration may offer structural opportunities to normalize abortion, some participants worried it would dilute the abortion‐specific expertise and stigma‐reducing environment found in specialist services that had been critical to their experience of high‐quality care. This dynamic was evident in the way participants described standalone services. Some appreciated a specialized space with dedicated expertise and emotionally supportive care. Others viewed their separation from mainstream care as a symbol and source of stigma. This aligns with broader discussions on abortion exceptionalism and abortion stigma, which highlight how the separation of abortion from mainstream care can both perpetuate stigma and, paradoxically, protect patients from judgmental attitudes found elsewhere in the health system (Footman 2025). In this way, abortion services, in the various configurations in which they are provided, might be considered as simultaneously resisting and reproducing stigma.

Our findings support conceptualizations of normalization as a socially and emotionally mediated process. For some participants, care that was explicitly affirming and nonjudgmental made abortion feel “normal” despite its structural exceptionalism. In the United Kingdom, Maxwell et al (2021) found abortion providers employed various strategies to resist stigma and normalize abortion care both on behalf of themselves and their patients, including presenting abortion to both patients and colleagues as “unexceptional, routine healthcare”. They call for change at a structural level, however, not relying on individual providers to do all the work to normalize and destigmatize. This call is echoed in research in England and Wales that identifies how abortion is structurally stigmatized within the healthcare system in multiple ways, including through its separation from mainstream health services (Footman 2025). This is in contrast to Scotland, where care is delivered by the NHS in community SRH or in obstetrics and gynecology departments.

Our findings suggest that expanding the range of settings from which abortion care can be accessed has potential to contribute to this structural change by repositioning abortion care as essential reproductive healthcare. In theory, this has potential to disrupt the stigma produced by prevailing models of service provision (Link and Phelan 2001, 2014; Tyler and Slater 2018). However, findings also highlight an ambivalence and tension that abortion exceptionalism engenders: while many participants welcomed the idea of integration into primary care—especially in terms of improving access, normalizing abortion, and facilitating holistic care—they also feared losing the specialist, affirming ethos of abortion specialists. This tension reflects a well‐documented dilemma in abortion service design: integration may help reduce stigma and improve continuity of care, but without proper training and system redesign, it risks exposing people to generalist settings that are unprepared to meet their needs or protect them from judgment (Footman 2025) if stigma continues to be produced through other means (Link and Phelan 2001, 2014; Tyler and Slater 2018), for example a lack of empathetic care through insufficient investment in provider training in generalist settings. Care that was explicitly reassuring, empathic and nonjudgmental, that is, care that *accounted* for the stigma around abortion and its exceptionalized nature, contributed to participants experiencing their abortion as a normal (unexceptional) life event, including among those for whom it was a challenging process to undergo (Purcell et al. 2020). This points to the chicken‐and‐egg nature of normalization, whereby it is not a linear outcome but an ongoing, iterative process, which can be conceptualized as a “two‐way street” (Parker, Williams, and Aldridge 2002) that is shaped by both service design and social norms. Abortion is stigmatized *because* it is exceptionalized, and it is exceptionalized *because* it is stigmatized.

Our findings bring into relief the way abortion stigma and abortion exceptionalism are interlinked and mutually reinforcing. Participants’ responses highlighted how the stigma they expected to experience led them to seek safety in attending standalone services staffed by specialist providers, but also that separating abortion from mainstream services marked it out as different and contributed to its stigmatization. As Tyler and Slater (2018) note, stigma operates through policy, law, health system design, and cultural norms to regulate access to resources, rights, and legitimacy. So while structural change, like integrating abortion care into primary care settings, has potential to disrupt this cycle and contribute to destigmatization and normalization of abortion, this process will take time. Care that accounts for abortion's exceptionalized status remains critical to “bridge the gap” between what might be described as an optimal scenario, where abortion is recognized and experienced as unexceptional, commonly experienced healthcare (over 200,000 took place in England and Wales in 2022; Department of Health and Social Care 2025), and a realistic one, in which it remains an event that can be experienced as stigmatized and exceptionalized.

Our participants’ reflections on how configuration of abortion services shapes quality of care provided a strong policy steer in terms of the need for availability of choice of abortion care setting to accommodate the different needs of different people in different circumstances. While choice in relation to abortion has more often been discussed in the context of method (Baraitser et al. 2022; Footman 2024), our study demonstrates the importance of choice in healthcare setting. This echoes a growing policy emphasis on patient‐centered care and aligns with current guidance from the Royal College of Obtetricians and Gynaecologists (RCOG) (RCOG abortion taskforce 2022) and NHS England (2025), which advocate for expanded choice in abortion care, including in setting. Our findings suggest that by expanding the range of options available to people seeking abortion care, the extension of choice into healthcare setting can improve quality of care by enhancing access to and continuity of care, facilitating more holistic, person‐centered care, addressing stigma and challenging abortion exceptionalism by embedding abortion into the fabric of primary care. This is already in place in Scotland through provision in NHS hospitals and community SRH, and to an extent in the independent sector, where services are sometimes provided on (although separately to rather than integrated into) primary care premises, for example, an NHS hospital or GP surgery.

Our findings that participants valued choice of setting lend further weight to calls for full decriminalization of abortion in Britain (RCN 2024; RCOG and FSRH 2022). Decriminalization would remove the legal requirements for abortion to be approved by two doctors and take place on an approved premises and by a registered medical practitioner, parameters that constrain how abortion care can be provided and currently limit its provision in primary care settings. In doing so, it would challenge some of the institutional roots of abortion stigma that Tyler and Slater identify, namely the laws and policies that regulate and restrict care through mechanisms of social control. Legal constraints, including requiring approval from two doctors and limiting provision to permitted premises, not only reduce flexibility in care models but also institutionalize stigma (Bloomer and O'Dowd 2014; Sheldon 2016).

Our study highlights several further areas that would need to be addressed should abortion provision be integrated into primary care in Britain. The first is the need for more effective streamlining of services (Footman 2024). Continuity was considered a weakness of stand‐alone services, but those participants who did use hospital services did not appear to have fared a great deal better in terms of engagement with several agencies. Integrated care systems, with their remit for local service planning and cross‐sector coordination, could be key enablers of joined‐up abortion care (Health Foundation 2022). Collaborations with local public health commissioners, particularly in SRH, may offer further opportunities for integration. Workforce training is another critical component. Integration into primary care must be supported by education and training—both clinical and psychosocial—for all health professionals involved in abortion care. Lessons here can be learned from the Scottish model, where abortion provision in community SRH is common, and from other international settings that already allow GP provision. Canada and Australia have recently seen significant expansion of abortion provision into primary care (Mazza et al. 2022; Munro et al. 2020; Singh et al. 2023), and in France, abortion medication can be prescribed by both general practitioners and midwives and dispensed by pharmacists (Faucher 2021). The workforce training piece must include incorporating abortion teaching in medical, nursing, midwifery, and pharmaceutical education from the undergraduate level to have potential to reshape institutional cultures that currently treat abortion as exceptional or peripheral. Finally, attention to broader health system structures is essential. In Scotland, where there is no independent sector provision, many patients must travel to England to obtain an abortion after 16–20 weeks of gestation (Cameron et al. 2016; Grieve, Harden, and Boydell 2025; Purcell et al. 2014). Equitable access across gestations and geographies must be part of any integration strategy. Collaboration between NHS and independent providers could help facilitate some of these changes, including training opportunities, as well as enabling greater trust and smoother referral pathways between sectors (Footman 2025).

Our study sample was drawn from across Britain, from a range of providers—independent and NHS, and from multiple settings—standalone independent sector services, NHS hospitals, and community SRH clinics. We captured a range of procedures and recruited participants from different ethnic and socioeconomic backgrounds and of varied ages. This breadth strengthens the study's contribution to understanding the experience of abortion under different models of care. Future research that interrogates in more depth how abortion seekers’ demographic and socioeconomic characteristics shape their experiences of abortion under different models of care would help understand the implications of individuals’ backgrounds and present circumstances on the care choices they need. This should include questions of how views of different models of care are shaped by rurality or remote living to inform the design of services in such settings (where specialist services are more challenging to provide).

A limitation relates to the fact that participants’ views on the relative merits of different models of care were, in large part, hypothetical, particularly those on pharmacy and GP provision (and provision in community SRH by participants in England and Wales). Views on these models were therefore shaped more by expectation than direct experience. The fact that participants had only experienced abortion provision in permitted premises and that satisfaction levels were generally high, may have skewed their preferences towards the model of care received. That said, research (e.g., in the United Kingdom, Ireland, North America, and Australia) has documented stigmatizing encounters in sexual and reproductive healthcare among pharmacy and GP staff including unsupportive or indiscreet attitudes, echoing the hypothetical claims of our participants that were often also based on their own experiences of attending such services for other reasons (Sweeney et al 2015; Assifi et al. 2025; O'Shea et al. 2020). The majority of participants had had early medical abortions, a smaller number of participants had later gestation or surgical abortions, and the full range of their views on the merits of different models of care may not have been captured in our sample. Participants’ awareness of health systems aspects of different models of abortion care will likely be limited. These understandings may shape what they perceive to be feasible or desirable models of care. Still, their reflections are valuable in revealing how abortion exceptionalism is experienced and interpreted, and how stigma—both at an individual level (expectations and previous experiences) and structural level (particularly service design, but also legal, policy and health system factors)—shapes expectations and care experiences. This study examined alternative models of provision within formal health systems—research interrogating views on provision outside of the clinic or medical setting would further add to our understanding of patient preferences for their abortion care. Our analysis permitted us to draw on the advantages of both a deductive and inductive approach to coding (Robinson et al. 2026; Fife et al. 2024). The approach allowed us to structure the findings around an existing theoretical framework (quality of care) to ensure relevance to policy and practice whilst bringing in wider theory (on stigma and abortion exceptionalism) and being open to insights outside of the preidentified themes (e.g., the significance of the intersection between provider and place, and the central role of choice and autonomy in where and how abortion care is accessed as factors in how it is experienced). However, this practice‐focused approach may have led us to miss themes that did not sit within the broad deductive categories identified prior to analysis.

Integration, if done well, can contribute to destigmatization of abortion and move it closer to being recognized and experienced as unexceptional, essential care. However, a central insight from our findings is that integration should not be framed as a simple administrative or logistical shift, but as a deliberate effort to reposition abortion as essential reproductive healthcare, while actively addressing the complexities that currently shape abortion experiences, including experiences of stigma. This repositioning of abortion requires both the transformation of structural conditions (laws, service delivery, institutional cultures) and attention to the needs of patients navigating stigma in real time. Integrated care must therefore be flexible, inclusive, and person‐centered, ensuring that every person can access abortion in the setting that best meets their needs.

## AUTHOR CONTRIBUTION

RS: Conceptualization, Formal analysis, Funding acquisition, Investigation, Methodology, Writing – original draft; ML: Investigation, Methodology, Writing – review and editing; RM: Investigation, Methodology, Writing – review and editing; NS: Investigation, Methodology, Writing – review and editing; PL: Funding acquisition, Methodology, Writing – review and editing; SC: Funding acquisition, Methodology, Writing – review and editing; JR: Investigation, Writing – review and editing; MP: Methodology, Writing – review and editing; RF: Conceptualization, Funding acquisition, Methodology, Writing – review and editing; KW: Conceptualization, Funding acquisition, Formal analysis, Methodology, Writing – review and editing

## ETHICS STATEMENT

This work was approved by LSHTM (Ref: 22761), NHS (IRAS Project ID: 291993), and BPAS (Ref: 2021/02/WEL). All participants gave informed consent prior to enrolment in the study.

## CONFLICT OF INTEREST STATEMENT

None to declare.

## Data Availability

Research data are not shared.
